# HSV as A Platform for the Generation of Retargeted, Armed, and Reporter-Expressing Oncolytic Viruses

**DOI:** 10.3390/v10070352

**Published:** 2018-06-30

**Authors:** Laura Menotti, Elisa Avitabile, Valentina Gatta, Paolo Malatesta, Biljana Petrovic, Gabriella Campadelli-Fiume

**Affiliations:** 1 Department of Pharmacy and Biotechnology, University of Bologna, Bologna 40126, Italy; laura.menotti@unibo.it (L.M.); elisa.avitabile@unibo.it (E.A.); 2 Department of Experimental, Diagnostic and Specialty Medicine, University of Bologna, Bologna 40126, Italy; valentina.gatta6@unibo.it (V.G.); biljana.petrovic2@unibo.it (B.P.); 3 Department of Experimental Medicine, University of Genoa, Genoa 16132, Italy; paolo.malatesta@unige.it; 4 Ospedale Policlinico San Martino—IRCCS per l’Oncologia, Genoa 16132, Italy

**Keywords:** retargeting, oncolytic herpesvirus, PSMA, EGFR, EGFRvIII, mIL12, Gaussia Luciferase, HER2

## Abstract

Previously, we engineered oncolytic herpes simplex viruses (o-HSVs) retargeted to the HER2 (epidermal growth factor receptor 2) tumor cell specific receptor by the insertion of a single chain antibody (scFv) to HER2 in gD, gH, or gB. Here, the insertion of scFvs to three additional cancer targets—EGFR (epidermal growth factor receptor), EGFRvIII, and PSMA (prostate specific membrane antigen)—in gD Δ6–38 enabled the generation of specifically retargeted o-HSVs. Viable recombinants resulted from the insertion of an scFv in place of aa 6–38, but not in place of aa 61–218. Hence, only the gD N-terminus accepted all tested scFv inserts. Additionally, the insertion of mIL12 in the US1-US2 intergenic region of the HER2- or EGFRvIII-retargeted o-HSVs, and the further insertion of Gaussia Luciferase, gave rise to viable recombinants capable of secreting the cytokine and the reporter. Lastly, we engineered two known mutations in gB; they increased the ability of an HER2-retargeted recombinant to spread among murine cells. Altogether, current data show that the o-HSV carrying the aa 6–38 deletion in gD serves as a platform for the specific retargeting of o-HSV tropism to a number of human cancer targets, and the retargeted o-HSVs serve as simultaneous vectors for two molecules.

## 1. Introduction

Oncolytic virotherapy and immunotherapy represent a novel frontier in cancer treatment. The therapeutic viruses can be combined with standard of care or novel treatments, including check point inhibitors, for front-line or second-line treatments [1,2,3,4,5,6]. A variety of viruses are being evaluated as anti-cancer agents in clinical trials. In a few cases, the wild type (wt) or naturally occurring mutant forms were employed. In most cases, genetic modifications were introduced in order to confer cancer specificity [7,8,9,10,11]. Genetically engineered and mutant forms of herpes simplex virus (HSV) stand as effective or as highly promising agents because of a number of features, including the ample genome (150 kbp) capable of accepting the insertion of heterologous genes, and the detailed knowledge of the properties of the viral genes, with respect to virus biology and pathogenesis (reviewed in [7]). The oncolytic HSV (o-HSV) named Oncovex^GM-CSF^ (granulocyte monocyte-colony stimulating factor) or T-VEC (Talimogene laherparepvec, commercial name Imlygic), approved for the therapy of metastatic melanoma by the Food and Drug Administration and European Medicines Agency [12,13,14,15,16,17], and the o-HSVs in clinical trials gain cancer specificity from genetic modifications or natural mutations which attenuate virulence and replication to varying degrees [18,19,20,21,22]. Not all these mutants are highly effective, and there is a need for o-HSVs which replicate robustly in cancer cells and are highly cancer specific. To meet these needs, we and others designed non-attenuated o-HSVs retargeted to cancer specific receptors of choice, exemplified by IL13Rα2 (Interleukin 13 receptor α2), HER2 (human epidermal growth factor receptor 2), EGFR (epidermal growth factor receptor), and uPAR (urokinase plasminogen activator receptor) [23,24,25,26,27,28,29]. The retargeted viruses carry no genomic modifications other than those required for the tropism retargeting; hence, they are fully virulent.

The first-generation retargeted o-HSVs carry the retargeting ligand in gD, in place of a portion of the glycoprotein. The deleted sequence is critical for gD interaction with the natural receptors HVEM (herpesvirus entry mediator) and nectin1. The resulting recombinants are detargeted from these receptors [30]. In our laboratory, the selected cancer target was human HER2 and the retargeting moiety was a high-affinity scFv. The HER2-retargeted viruses named R-LM113 and R-LM249 carry the deletion of aa 6–38 or of aa 61–218, respectively [27,28]. They exhibit safety and efficacy in preclinical models of immunosuppressed or immunocompetent mice. The routes of administration have been intra-tumoral, intra-peritoneal, or systemic via carrier cells [28,31,32,33,34]. Glorioso and Grandi selected EGFR for the successful design of retargeted HSVs optimized for glioblastoma therapy [29]. Recently, we demonstrated that gH or gB, two additional glycoproteins essential for HSV entry, can also serve as retargeting tools [35,36]. The availability of alternative retargeting strategies led to the construction of a non-cancer cell line for the in vitro cultivation of retargeted o-HSV [37]. In particular, the recombinants carrying the scFv to HER2 in gD and the GCN4 peptide in gB, gH, or gD infected and replicated in a Vero cell line derivative named Vero-GCN4R, which transgenically expresses an artificial receptor to GCN4, named GCN4R [37,38,39]. The double-retargeted recombinants alternatively infect the HER2-positive cancer cells via HER2, and the producer Vero-GCN4R cell line via the GCN4-GCN4R interaction. Additional strategies to attain cancer specificity include transcriptional retargeting [40] and retargeting through miRNAs differentially expressed in healthy or tumor tissue [41], exploiting the pathway of miRNAs which are abundantly expressed or induced across the herpesviridae family [42].

The aims of this work were threefold: (i) to ascertain whether we could generate o-HSVs retargeted to tumor cell specific receptors other than HER2 by using the backbones of R-LM113 or of R-LM249. The selected targets were EGFR, in its wt form or variant III form (EGFRvIII), and the prostate specific membrane antigen (PSMA), expressed in a variety of cancers, in glioblastoma and in prostate cancers, respectively; (ii) to identify additional sites in the HSV genome for the insertion of heterologous genes, exemplified by the interleukin 12 (IL12) cytokine, and to test the simultaneous expression of the novel reporter Gaussia Luciferase (GLuc), suitable for viral detection in vitro and in vivo; and (iii) to investigate whether two previously described mutations in gB result in o-HSVs with enhanced spread in murine cells.

## 2. Materials and Methods

### 2.1. Cells and Viruses

We employed an array of different cell lines, taking into account the different receptor usage of the retargeted recombinant viruses. SK-OV-3 (human ovary adenocarcinoma), LNCaP (human prostate adenocarcinoma), and RS (rabbit skin) were obtained from ATCC. R6, a cell line expressing HSV gD in an inducible manner, was described in [43]. Rh4 rhabdomyosarcoma cells were described in [44]. U251 MG cells (human glioma, for simplicity designated as “U251” in this paper) were received from Dr. Bernard Roizman (University of Chicago). hGic-G7 and hGic-G15, human glioma initiating cells from glioblastoma patients, were described in [45]. MDA-MB-231 cells (EGFR positive mammary gland/breast metastatic adenocarcinoma), were received from Dr. Pier-Luigi Lollini (University of Bologna). CHO-EGFR cells were received from Dr. Steve Russell (Mayo Clinic, Rochester, MN, USA) [46]. TRAMP-PSMA and PC3-PIP-PSMA cells were received from Dr. Marco Colombatti and Dr. Giulio Fracasso (University of Verona). J cells, resistant to HSV infection, and J-nectin1, expressing the single human receptor nectin1, were described in [47]. J-HER2 cells were described in [26]. Here, J cells expressing EGFR, EGFRvIII, or PSMA were generated by transfection of the plasmids described below (see Section 2.2). U251-EGFRvIII cells were generated by plasmid transfection and cell cloning, while B16-HER2 cells (mouse melanoma) were generated by transduction with a lentiviral vector [48]. The expression of heterologous receptors in cell lines was checked by flow cytometry. Recombinant BACs and viruses described in this paper are listed in Table 1; the cell lines used for their characterization are listed in Table 2. The cell lines employed for virus titrations are listed in Table 3.

HSV-1(F) is the wt prototype [49]. R-LM5 carries *loxP*-bracketed pBeloBAC11 (which includes BAC sequences) between UL3 and UL4 [50] and the reporter EGFP gene engineered in pBeloBac11. It is the wt-gD counterpart of the HSV recombinants [27]. R-LM113 and R-LM249 carry an scFv to HER2 in gD Δ6–38 or gD Δ61–218, respectively, in addition to the BAC sequences inserted between UL3 and UL4, as in R-LM5 [27,28]. The key features of viruses are listed in Table 1. Virus stocks were prepared by infecting cell monolayers in T175 flasks with virus seeds at a multiplicity of infection (MOI) of 0.5 PFU/cell and harvesting at 48 h. Infected cells were scraped and pelleted at 300× *g*. Pellets were sonicated for 10 s on ice, and frozen at −80 °C in 50 μL aliquots to avoid repeated freeze-thaw cycles. One aliquot for each virus stock was thawed to determine the titer in the cell lines listed in Table 3. For titrations, cells were infected with serially diluted virus stocks. After 1.5 h absorption, the monolayers were overlaid with medium containing agarose (1% Seaplaque Agarose). Plaques were counted at day 3.

### 2.2. Plasmids

The following plasmids were generated, or obtained from other laboratories, in order to engineer cell lines expressing cancer-specific receptors. pIRES-PSMA was obtained from Angelo Baccala, Cleveland Clinic. pcDNA-EGFR I (isoform “a”), the cDNA encoding EGFR, was amplified with primers T7 and BGHrev using the DNA from CHO-EGFR cells as a template. The amplicon was cut with XbaI and HindIII and cloned in pcDNA3.1(-). The plasmid pCDNA-EGFRvIII was engineered by performing a deletion in the pcDNA-EGFR I isoform “a” insert, with primers BGHrev and Xba_EGFRvIII_f (TGT GCT CTA GAT GCG ACC CTC CGG GAC GGC CGG GGC AGC GCT CCT GGC GCT GCT GGC TGC GCT CTG CCC GGC GAG TCG GGC TCT GGA GGA AAA GAA AGG TAA TTA TGT GGT GAC AGA TCA CGG). The latter is a chimeric primer including the XbaI site, the sequence encoding the signal peptide and the first five amino acids of mature EGFR, the GGT triplet (encoding the “scar” glycine resulting from the deletion of exons 2 to 7), and 23 nt annealing to the sequence encoding amino acids 274–280. As above, the amplicon was cut with XbaI and HindIII and cloned in pcDNA3.1(-). The inserts were sequenced for accuracy. Single chain antibodies (scFv) inserts: pSL1180-scFv-PSMA encoding J591 scFv directed to PSMA was obtained from Dr. Michel Sadelain, Memorial Sloan-Kettering Cancer Center; anti-EGFR scFv, cloned in pTNHaa-EGFR, was obtained from Dr. Steve Russell, Mayo Clinic [46]; and anti-EGFRvIII scFv (cloned in plasmid pMR1ENV1), was obtained from Dr. Ian Lorimer, Ottawa Health Research Institute [57]. The plasmids were used as a template to amplify the respective scFvs, which were subsequently inserted in the appropriate recombinant HSV genome by means of galK recombineering [35,58]. The primers employed for amplification are listed in Table 4.

The mIL12 expression cassette was assembled in several steps. First of all, the ORF of mIL12b (subunit b) from clone IMAGE 40046200 was cloned in pcDNA3.1(-) downstream of a synthetic Kozak sequence with primers mIL12b_f (CAG ACC AGG CAG GCG GCC GCA CGC CAC CAT GTG TCC TCA GAA GCT AAC CAT CTC) and mIL12b_Eco_r (AAC GTT GAA TTC TAG GAT CGG ACC CTG CAG GGA), generating the pLM82 plasmid. Next, the ORF of mIL12a (subunit a) was inserted downstream of mIL12b ORF, following amplification from clone IMAGE 40126170 with primers mIL12a_Bam_f (TGC CGG ATC CGC ATG TGT CAA TCA CGC TAC CTC CTC) and mIL12a_Hind_r (AGG GCC TTG AAG CTT TCA GGC GGA GCT CAG ATA GCC C), generating the pLM83 plasmid. To ensure the expression of both subunits, an IRES sequence was amplified from pIRESneo with primers IRES_Eco_f (GTC TAG AAT TCA TTT TCC ACC ATA TTG CCG TCT TTT GG) and IRES_Bam_r (TAT TGG ATC CAG GAA TTA TCC CGG GGT TGT GGC AAG C), and cloned between the two mIL12 subunits, thus generating the final plasmid pLM84. pCMV-GLuc2 containing the copepod Gaussia princeps Luciferase under the control of the pCMV promoter was obtained from New England Biolabs. Plasmid gB_D285N_A549T cl 2 was generated by a site directed mutagenesis kit of the gB ORF cloned in pcDNA3.1(-), by means of QuickChangeII XL Stratagene with primer gB_D285N_EcoRI_f (GCT CGG TGT ACC CGT ACA ACG AAT TCG TGC TGG CGA CTG GC) and its complement, and primer gB_A549T_NheI_f (GCA AGC TGA ACC CCA ACA CGA TCG CTA GCG CCA CCG TGG GCC GGC GGG) and its complement.

### 2.3. Engineering of the Recombinant HSVs

The recombinant viral DNAs containing the BAC sequences, and generated by genetic engineering in bacteria, are hereafter designated as “BAC”, followed by the appropriate number. The recombinant viruses are designated with the letter “R”, followed by the appropriate number. To exemplify, when the recombinant BAC-593 is transfected into appropriate cells, it generates the corresponding HSV-1 recombinant named R-593.

#### 2.3.1. Structure of the HSV Backbone

HSV BAC genomes were engineered by galK recombineering [35,58]. In the intergenic UL3 and UL4 region, all the recombinant genomes carry the *loxP*-bracketed pBeloBac11, which includes the BAC sequences [50], and the reporter EGFP gene [27,28]. Glycoprotein D carries either the deletion of amino acids 6–38 or 61–218. Some derivatives were further modified to express mIL12 or Gaussia Luciferase (GLuc). The main characteristics of the recombinants are summarized in Table 1. Table 4 summarizes the templates and primers used to generate each construct. Specific modifications for retargeting purposes were as follows. The recombinants containing the scFvs in place of aa 6–38 were R-593 (scFv to PSMA), R-613 (scFv to EGFRvIII), and R-611 (scFv to EGFR). Downstream of the scFvs, we inserted linker L3 (SSGGGSGSGGS). The BACs containing the scFvs in place of aa 61–218 were BAC-591 (scFv to PSMA), BAC-623 (scFv to EGFRvIII), and BAC-621 (scFv to EGFR); the scFv was flanked by upstream linker L1 (HSSGGGSG) and by downstream linker L2 (SSGGGSGSGGSG).

#### 2.3.2. Mutagenesis of gB

BAC-LM249 was the starting genome to generate BAC-291, which carries the mutations D285N and A549T in gB [59]. A fragment encompassing the mutations was used to perform galK recombineering in gB ORF (Table 4).

#### 2.3.3. Insertion of mIL12 Cassette

As a site of insertion for mIL12, we chose the intergenic region between US1 and US2. The insertion was in the middle of the US1-US2 intergenic region, at coordinate 133854 (HSV-1 strain F, GenBank # GU734771.1), 84 bp downstream of the US1 ORF stop codon.

#### 2.3.4. Production of Recombinant Viruses

Recombinant viruses were reconstituted by transfection of the purified BAC genome in mammalian cells (R6) and subsequently amplified in cell lines expressing the appropriate target receptor: R-593 in J-PSMA and R-613 in J-EGFRvIII or U251-EGFRvIII. R-611 was initially reconstituted in CHO-EGFR, and then passaged in RS or in SK-OV-3 cells. The same approach was applied to BAC-591, BAC-623, and BAC-621, but it did not yield a recombinant virus able to replicate in cells expressing the cognate target receptor.

### 2.4. Viral Tropism

Cell monolayers were exposed to the recombinant viruses at MOI ranging from 2 to 10 PFU/cell. Infection was monitored through EGFP by fluorescence microscopy. Flow cytometry (BD Accuri C6) was used to measure EGFP or the binding of MAb 52S to gH (see Supplementary Materials). Neutralization of infection by cetuximab (Merck) was assayed by incubating cells with 83 µg/mL cetuximab 1 h before, during (1.5 h), and after (24 h) exposure to the virus.

### 2.5. Virus Growth

Cells seeded in 12-well plates were infected at 0.1 or 1 PFU/cell. After 90 min of virus adsorption, the inoculum was removed and the unpenetrated virus was inactivated by an acid wash (40 mM citric acid, 10 mM KCl, 135 mM NaCl, pH 3) [60] and cultures were frozen at 24, 48, or 72 h after infection. The progeny virus (intracellular plus extracellular) was titrated in a receptor-positive cell line (see Table 3), as detailed above in Section 2.1. The effect of cetuximab (Merck) on virus growth was assayed as described in Section 2.4; samples were collected at 24 and 48 h and titrated in receptor-positive cells. Data were plotted as the average of at least two independent experiments ± SD, as detailed in Figure legends.

### 2.6. Viral Spread Assay

Cells were infected with serially diluted virus stocks. After 1.5 h of adsorption, the monolayers were covered with an agarose overlay (1% Seaplaque Agarose). Infection and plaque formation were monitored over three days. For plaque size measurement, 35 pictures of plaques for each sample were taken with a Nikon DS-Vi1 camera, connected to a Nikon fluorescence microscope, and equipped with a 4× objective. Plaque area in pixels was measured with the ImageJ “Measure” tool and used to perform a statistical analysis (*T*-Test, two-tailed, Significance Level: 0.05).

### 2.7. Cytotoxicity Assay

Cells in 96 well plates (8·10^3^ cells/well) were infected at the indicated MOI or mock-infected. AlamarBlue (10 μL/well, Life Technologies, Carlsbad, CA, USA) was added to the culture media at two to eight days after infection. Four hours later, plates were read with a GloMax Discover System (Promega, Madison, WI, USA) or with an EnSpire Multimode Plate Reader (PerkinElmer, Waltham, MA, USA), according to the manufacturer’s instructions. Cell viability was expressed as the percentage difference in alamarBlue reduction in infected relative to uninfected cells. Samples were run at least in triplicate and the results were plotted as average ± SD.

### 2.8. ELISA for mIL12

Cells were seeded in 48-well plates and infected with recombinant viruses. The concentration of mIL12 secreted in the medium was measured with the Mouse IL12 (p70) ELISA Plate kit (ThermoFisher Scientific Pierce, Waltham, MA, USA), according to the manufacturer’s instructions. Absorbance was read with a GloMax Discover System (Promega) at 450 and 560 nm.

### 2.9. GLuc Assay

The luminescence activity of GLuc secreted by cells infected with recombinant viruses was determined by incubating 25 μL of the cell medium (diluted 1:3 in DMEM) with 50 μL of Dual-Glo Stop & Glo Substrate (Promega), shaking for 5 s, and reading with a GloMax Discover System (Promega) with 1 s of integration time. Gaussia Luciferase activity is plotted as RLU after normalization to the background of the viral inoculum. The graphs represent the average of three independent experiments ± SD.

## 3. Results

### 3.1. Construction of Cell Lines Expressing the Targeted Receptors PSMA, EGFRvIII and EGFR

**Figure 1:**
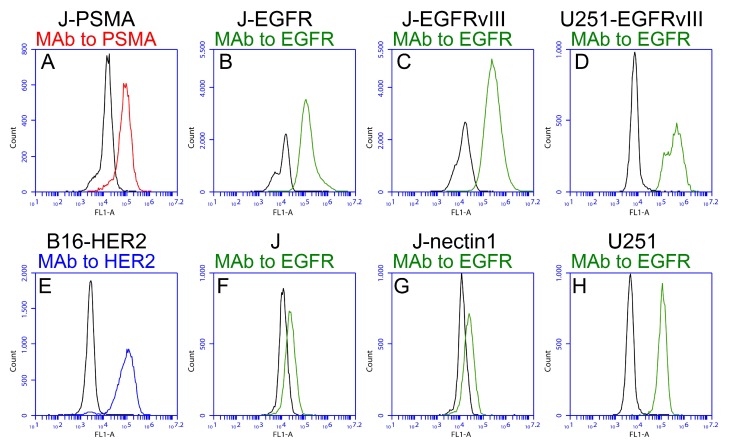
Expression of the indicated receptors in the cell lines generated and employed in this study. Already available cell lines are included for comparison. Cells were reacted with monoclonal antibodies to PSMA (**A**, red line), EGFR (**B**–**H**, green line), or HER2 (**E**, blue line). MAb to EGFR is reactive to both EGFR and EGFRvIII. Black lines represent the background signal of the secondary antibody alone.

In order to analyze the tropism of the recombinants described in this study, we generated a number of cell lines expressing a single heterologous tumor cell specific receptor. Thus, J cells negative for HSV-1 natural receptors [47], were engineered to express PSMA, EGFR, or EGFRvIII. The expression of the targeted receptors was checked by flow cytometry (Figure 1A–C). EGFRvIII was transgenically expressed in the human glioma cell line U251, thus generating U251-EGFRvIII (Figure 1D). Of note, the double peak in pane l D accounts for the expression of wt EGFR in U251, as shown in Figure 1H. The mouse melanoma cell line B16 was engineered to express HER2 (Figure 1E). The reactivity of the MAb to EGFR on J and J-nectin1 cell lines documents the expression of a rodent ortholog of EGFR (Figure 1F,G).

### 3.2. The PSMA-Retargeted R-593

**Figure 2:**
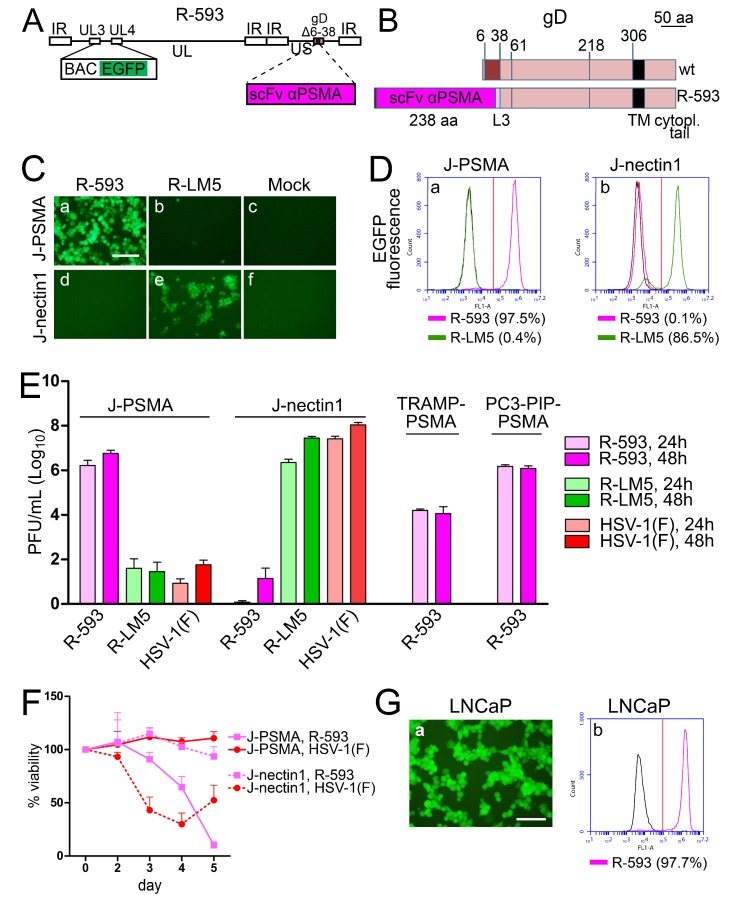
Properties of PSMA-retargeted R-593. (**A**) Schematic drawing of the R-593 genome. Inverted repeat (IR) sequences are represented as rectangular boxes. The scFv to PSMA is inserted in gD, in place of aa 6–38. pBeloBAC11 and EGFP (BAC EGFP) are inserted between UL3 and UL4 [50]. (**B**) Schematic linear maps of R-593 gD, and of wt gD. The aa coordinates relevant to this study are indicated. The two linear maps are drawn to scale. Scale bar, 50 amino acid residues. Note that the size of R-593 gD is almost doubled relative to that of wt gD, as a consequence of scFv insertion. L3, linker 3. (**C**) J-PSMA or J-nectin1 cell lines were infected with the indicated viruses at 10 PFU/cell. Pictures were taken at 24 h p.i. The levels, brightness, and contrast of the panels were adjusted by means of Adobe Photoshop as follows: input level (black) 30, brightness +60, contrast +60, scale bar =100 µm. (**D**) J-PSMA or J-nectin1 cells exposed to 10 PFU/cell of R-593 or R-LM5 or mock infected (black line); at 24 h after infection, the infected cells were analyzed for EGFP fluorescence by flow cytometry. The figures next to each virus name indicate the percentage of infected cells. Vertical red line: threshold discriminating the negative and positive populations, as determined by comparison with the negative control. (**E**) R-593 replication in J-PSMA, J-nectin1, or the PSMA-expressing prostate murine (TRAMP-PSMA) or human (PC3-PIP-PSMA) tumor cell lines. Cells were infected at 0.1 PFU/cell, and harvested at 24 or 48 h after infection. The plotted values represent the average of three independent experiments (for J-PSMA and J-nectin1) or two independent experiments for TRAMP-PSMA and PC3-PIP-PSMA, ± SD. (**F**) Effect of virus infection on viability of J-PSMA (solid lines) or J-nectin1 (dashed lines) cells, infected at 2.5 PFU/cell. The data represent the average of four replicate samples ± SD. (**G**) R-593 infection of the human prostate cancer cell line LNCaP detected by fluorescence microscopy (panel a). Panel b shows the extent of infection as detected by flow cytometry. Vertical red line: threshold between negative and positive populations, scale bar = 100 µm.

R-593 was designed to target the prostate tumor cell specific receptor PSMA [61]. It was engineered by insertion of the anti-PSMA scFv in gD in place of aa 6–38, and therefore carries the same deletion in gD as R-LM113 (the schematic diagram of R-593 genome and the linear map of wt and R-593 gD are shown in Figure 2A,B). The aa 6–38 deletion in gD ablates targeting and binding to nectin1 and HVEM [27]. The key features of R-593 are summarized in Table 1. In this and previous work, for retargeting assays, we preferentially employed J cells, a derivative of BHK-TK cells, resistant to HSV infection and defective in the expression of the natural HSV receptors [47], and a number of its derivatives transgenically expressing a single receptor of choice [26,27,28]. To exemplify, J-nectin1, J-HER2, and J-PSMA enable the infection of wt-HSV-1, the HER2-retargeted o-HSVs, or the PSMA-retargeted R-593, respectively. We also included R-LM5, a recombinant which carries the BAC sequences and the EGFP reporter in the UL3-UL4 intergenic region, and wt-gD [27], which is the wt counterpart of the recombinants (summary of properties in Table 1). Infection was monitored by fluorescence microscopy (Figure 2C), taking advantage of the EGFP reporter engineered in R-593, and was quantified by flow cytometry as EGFP fluorescence (Figure 2D), or by indirect fluorescence with MAb 52S to gH (Supplementary Table in Supplementary Materials). Figure 2C, panel a, and 2D, panel a, show that R-593 efficiently infected J-PSMA cells (97.5% by flow cytometry) and failed to infect J-nectin1 cells (Figure 2C, panel d; 0.1% infection by flow cytometry in Figure 2D, panel b). In contrast to R-593, R-LM5 efficiently infected J-nectin1 cells (Figure 2C, panel e, and Figure 2D, panel b; 86.5% by flow cytometry), and caused infection of J-PSMA cells only in a few cells in the monolayer (Figure 2C, panel b; 0.4% by flow cytometry, Figure 2D, panel a). Virus yield experiments showed that R-593 replicated to yields of 10^6^ PFU/mL in J-PSMA cells, and to yields of 10^2^ PFU/mL in J-nectin1 cells, corresponding to one progeny virion per about 1000 cells. Conversely, HSV-1(F) and R-LM5 replicated to yields of 10^6^ PFU/mL or higher in J-nectin1 cells, and to yields of 10^2^ PFU/mL in J-PSMA cells. The latter figure is consistent with the extent of EGFP detection in Figure 2C, panels b. Together, the tropism and the virus growth experiments in Figure 2C–E support the conclusion that R-593 was retargeted to PSMA and detargeted from the natural receptor nectin1, as expected, because of the aa 6–38 deletion in gD. We consider the few infected cells seen in Figure 2C, panel b, the flow cytometry extent of infection below 5%, and the yields around 10^2^–10^3^ PFU/mL as negligible background here and in subsequent experiments. It may be consequent to the so called unconventional infection of HSV-1 in J cells [59,62].

With respect to growth in cancer cell lines, R-593 replicated in PC3-PIP-PSMA, a cell line derived from a human prostate adenocarcinoma made transgenic for PSMA, to a titer similar to that attained in J-PSMA (Figure 2E). Replication in the murine prostate cancer TRAMP-PSMA was about two orders of magnitude lower (Figure 2E), a result in agreement with the scarce infection of murine cells, in particular cells derived from C57BL/6 mice, which are among the most highly resistant to HSV [63,64,65]. The cytotoxic activity of R-593 was specific for PSMA expressing cells, and was undetectable in J-nectin1 cells (Figure 2F). Conversely, HSV-1(F) exerted no cytotoxic effect in J-PSMA cells, which express PSMA as the sole receptor, but was cytotoxic in J-nectin1, as expected (Figure 2F). Of note, R-593 readily infected LNCaP cells, a human prostate cancer cell line, which naturally expresses PSMA (Figure 2G), implying that the extent of PSMA expression in human cancer cells makes them a target for R-593 infection and virotherapy. These results indicate that it is possible to specifically retarget the HSV tropism to the human PSMA prostate tumor cell specific receptor by engineering an scFv to PSMA in gD, in place of the 6–38 aa sequences, which are critical for the interaction with the gD receptors HVEM and nectin1.

### 3.3. The EGFRvIII-Retargeted R-613

**Figure 3:**
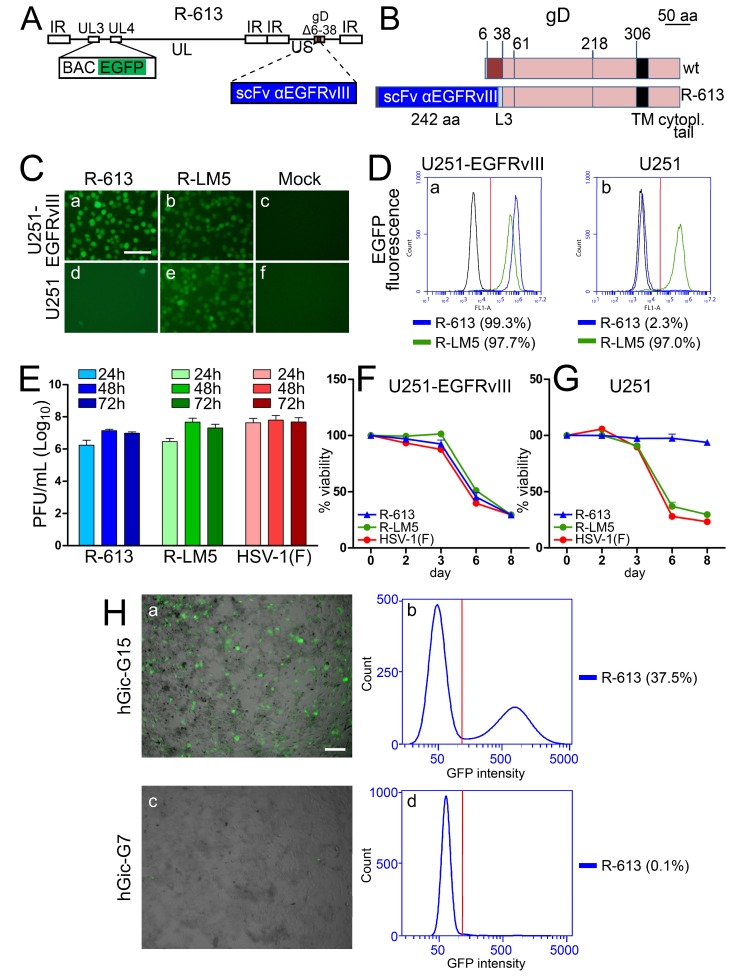
Properties of the EGFRvIII-retargeted R-613. (**A**) Schematic drawing of the R-613 genome. The scFv to EGFRvIII is inserted in place of aa 6–38 of gD. pBeloBAC11 and EGFP (BAC EGFP) are inserted between UL3 and UL4 [50]. (**B**) Linear maps of R-613 gD and of wt gD. The aa coordinates relevant to this study are indicated. The two linear maps are drawn to scale. Scale bar, 50 amino acid residues. L3, linker 3. (**C**) U251-EGFRvIII or U251 cell lines were infected with the indicated viruses at 10 PFU/cell, or mock-infected. Pictures were taken at 24 h p.i. The levels, brightness, and contrast of the panels were adjusted by means of Adobe Photoshop as follows: input level (black) 30, brightness +60, contrast +60, scale bar =100 µm. (**D**) U251-EGFRvIII or U251 cells exposed to 10 PFU/cell of R-613 or R-LM5, or mock infected (black line), were analyzed 24 h after infection by flow cytometry for EGFP. The figures next to each virus name indicate the percentage of infection. Vertical red line: threshold discriminating the negative and positive populations, as determined by comparison with the negative control. (**E**) R-613 replication in U251-EGFRvIII cells infected at 0.1 PFU/cell and harvested 24, 48, and 72 h after infection. The data represent the average of three independent experiments ± SD. (**F**,**G**) Effect of virus infection on cell viability. U251-EGFRvIII (F) and U251 (G) cells were infected at 2.5 PFU/cell. Viability was quantified at the indicated days after infection. The data represent the average of four replicate samples ± SD. (**H**) R-613 infection of the human glioma initiating cells hGic-G15 (a, b), but not of hGic-G7 (c, d). Scale bar = 100 µm. Figures next to panels b and d indicate the extent of infection as detected by cytofluorimetric analysis. Vertical red line: threshold between negative and positive populations.

The R-613 recombinant was designed to target EGFRvIII, a glioma-specific variant of EGFR which carries a 26 amino acid deletion in the extracellular domain [66,67,68]. R-613 was engineered by insertion of the anti-EGFRvIII scFv in gD in place of aa 6–38, and therefore carries the same deletion in gD as R-LM113 (the schematic diagram of the R-613 genome and the linear map of wt and R-613 gD are shown in Figure 3A,B). The key features of R-613 are summarized in Table 1. Given that EGFRvIII is a glioma-specific receptor, for tropism and virus growth assays, we made use of a human EGFRvIII-negative glioma cell line (U251), in which we transgenically expressed EGFRvIII (U251-EGFRvIII). The tropism assay showed that R-613 selectively infected U251-EGFRvIII but not wt U251 cells, as seen by fluorescence microscopy (Figure 3C, panels a and d) and quantified by flow cytometry (Figure 3D; 99.3% and 2.3% infection in U251-EGFRvIII and U251 cells, respectively). Thus, R-613 was retargeted to EGFRvIII and detargeted from nectin1. R-613 replicated efficiently in U251-EGFRvIII cells, and reached yields similar to those attained by R-LM5 (Figure 3E). In agreement with the virus growth and tropism assays, R-613 exhibited cytotoxic activity in U251-EGFRvIII cells (Figure 3F), but not in U251 cells (Figure 3G). To ascertain whether R-613 was capable of infecting human glioma cells, we employed cultures of human glioma initiating cells (hGic), also known as glioma stem cells, from glioblastoma patients [45]. By real time RT-PCR, the extent of EGFRvIII expression was quantified to be at least 250 fold higher in hGic-G15 than in hGic-G7, where it was undetectable over 40 PCR cycles; hence hGic-G7 and hGic-G15 were considered to be negative and positive for EGFRvIII, respectively (Figure S1 in Supplementary Materials). As shown in Figure 3H, hGic-G15 cells were infected with R-613 (panels a, b), whereas hGic-G7 cells were not (panels c, d). Cumulatively, the data indicate that for specific retargeting, the 6–38 aa sequences of gD can be successfully substituted with an scFv to EGFRvIII receptor, and that the extent of EGFRvIII expression in human glioma initiating cells makes the latter a target for R-613 infection and virotherapy.

### 3.4. The EGFR-Retargeted R-611

**Figure 4:**
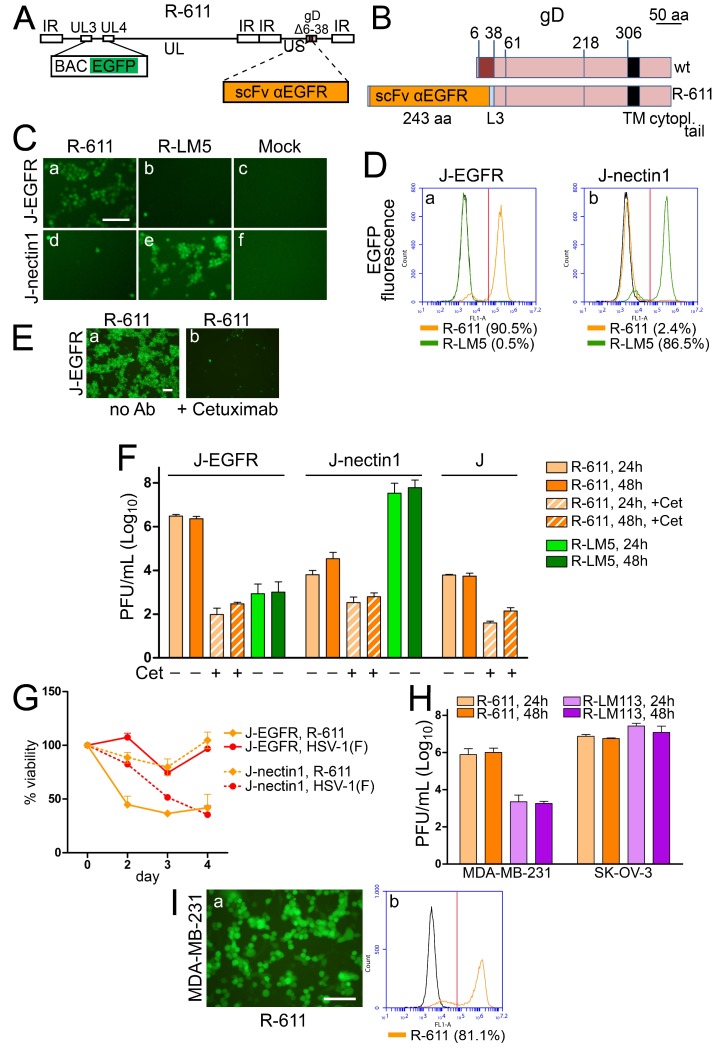
Properties of EGFR-retargeted R-611. (**A**) Schematic drawing of the R-611 genome. The scFv to EGFR was inserted in place of aa 6–38 of gD. pBeloBAC11 and EGFP (BAC EGFP) are inserted between UL3 and UL4 [50]. (**B**) Linear map of wt gD and of recombinant gD in R-611. L3, linker 3. (**C**) J-EGFR and J-nectin1 cells were infected with the indicated viruses at 10 PFU/cell. Pictures were taken at 24 h after infection. The controls in J-nectin1 cells (R-LM5 and Mock) were the same as shown in Figure 2C, since the experiments with different recombinant o-HSVs were run in parallel to facilitate comparison. The levels, brightness, and contrast of the panels were adjusted by means of Adobe Photoshop as follows: input level (black) 30, brightness +60, contrast +60, scale bar = 100 µm. (**D**) J-EGFR and J-nectin1 cells exposed to 10 PFU/cell of R-611 or R-LM5, or mock infected (black line), were analyzed by flow cytometry for EGFP fluorescence. The controls in J-nectin1 cells (R-LM5 and Mock) are the same as in Figure 2D. The figures next to each virus name denote the percentage of infection. Vertical red line: threshold discriminating the negative and positive populations, as determined by comparison with the negative control. (**E**) Neutralization of R-611 infection in J-EGFR cells by the humanized monoclonal antibody cetuximab directed to EGFR (83 μg/mL, panel b) or no antibody (no Ab) as control (panel a). Pictures were taken at 24 h after infection. Scale bar = 100 µm. (**F**) R-611 replication in J-EGFR, J-nectin1, and J cells, infected at 1 PFU/cell, in the absence or presence of cetuximab (Cet), and harvested at the indicated times. The data represent the average of three independent experiments ± SD. R-LM5 replication in J-EGFR and J-nectin1 cells, infected at 1 PFU/cell. The data represent the average of four independent experiments ± SD. (**G**) Effect of virus infection on cell viability. J-EGFR (solid lines) or J-nectin1 (dashed lines) cells were infected at 5 PFU/cell with the indicated viruses. The data represent the average of four replicate samples ± SD. (**H**) R-611 replication in EGFR-positive MDA-MB-231 and SK-OV-3 tumor cells infected with at 1 PFU/cell and harvested at 24 and 48 h. Bars represent the average of two independent experiments ± SD. (**I**) Infection of MDA-MB-231 cells was documented by fluorescence microscopy (panel a) and quantified by flow cytometry (panel b). Scale bar = 100 µm. Vertical red line: threshold between negative and positive populations.

R-611 was designed to target EGFR, a receptor overexpressed and/or amplified in a variety of cancers, their metastases, and also widely expressed in non-cancer cells [69,70,71]. R-611 was engineered by insertion of the anti-EGFR scFv in gD, in place of aa 6–38, and therefore carries the same deletion in gD as R-LM113 (see Figure 4A,B for a schematic diagram of the R-611 genome and the linear map of wt and R-611 gD). The key features of R-611 are summarized in Table 1. R-611 was reconstituted upon transfection of BAC-611 DNA in CHO-EGFR cells and grown in RS or SK-OV-3 cells. The tropism of the two batches was identical, ruling out that growth in one or the other of the two cell lines selected for a variant virus. We interpret the infection and growth in RS as possibly mediated by a rabbit ortholog of human EGFR. The results of the tropism assay in Figure 4C,D show that R-611 infected J-EGFR cells (Figure 4C, panel a; Figure 4D, panel a; 90.5% by flow cytometry) and infected J-nectin1 cells at background levels (Figure 4C, panel d; Figure 4D, panel b; 2.4% by flow cytometry). The retargeting to EGFR was confirmed by the neutralization of J-EGFR cell infection by the humanized monoclonal antibody cetuximab directed to EGFR (Figure 4E, panel b). R-611 replicated in J-EGFR cells (Figure 4F) to yields higher than 10^6^ PFU/mL, i.e., to yields similar to those achieved by the PSMA-retargeted R-593 in the J-PSMA cells. The infection was dramatically reduced by cetuximab. In contrast to R-593, R-611 replicated in J-nectin1 cells, at a level two orders of magnitude lower than that in J-EGFR cells. Such replication was independent of nectin1, since it was also observed in the parental receptor-negative J cells, and since, in both cells, it was reduced to background levels by cetuximab, hinting that it occurred in part through an EGFR ortholog. In support of this possibility, we observed a species non-specific reactivity of J and J-nectin1 cells to an MAb directed to human EGFR (Figure 1F,G). R-LM5 replicated to a background level in J-EGFR cells, and to very high yields in J-nectin1 cells. Cytotoxicity assays mirrored the infection pattern. Thus, R-611 efficiently killed J-EGFR but not J-nectin1 cells. Conversely, HSV-1(F) substantially spared J-EGFR cells, and was cytotoxic to J-nectin1 cells, as expected (Figure 4G). R-611 infected human EGFR-positive cancer cells (Figure 4H,I). Often, human cancer cells express different members of the EGFR family in varying combinations and degrees. This situation is exemplified by MDA-MB-231 and SK-OV-3 cells. The former express EGFR and low level HER2 [71]; the second express EGFR and a substantial level of HER2 [70]. Both cell lines enabled the replication of R-611 (Figure 4H). In addition the SK-OV-3 cells enabled the replication of the HER2-retargeted R-LM113, which replicated to background levels in MDA-MB-231. The data indicate that the aa 6–38 deletion in gD was suitable for engineering an scFv to EGFR, and enabled the generation of a viable EGFR-retargeted recombinant. Given the apparent wide distribution of EGFR in cancer and non-cancer cells, and its cross-specificity, the level of cancer-specificity that may be achieved through this receptor needs to be critically evaluated.

### 3.5. The Insertion of the scFvs to PSMA, EGFRvIII, or EGFR in the Backbone of R-LM249 Failed to Generate Viable Recombinants

**Figure 5:**
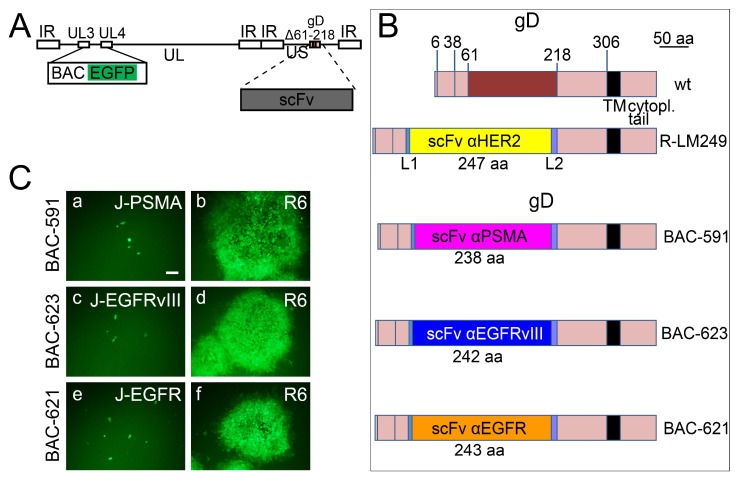
Failure to generate replication-competent recombinants by insertion of scFv in gD Δ61–218. (**A**) Schematic drawing of the recombinant genomes that carry the scFv in place of aa 61–218 of mature gD. pBeloBAC11 and EGFP (BAC EGFP) are inserted between UL3 and UL4 [50]. (**B**) Schematic linear maps of gD in recombinant BAC-591, BAC-623, and BAC-621 compared to wt gD. The aa coordinates relevant to this study are indicated. Scale bar, 50 amino acid residues. L1, L2, linker 1 and 2. (**C**) The recombinant genomes BAC-591, BAC-623, and BAC-621 carry non-functional recombinant gDs, and do not carry defects in genes other than gD. Transfection of BAC DNAs in R6 cells expressing wt-gD under the UL26.5 promoter allows the formation of plaques (panels b, d, f). Transfection of J cells expressing the individual target receptor only gives rise to single EGFP-positive cells (panels a, c, e). Pictures were taken at day 3 post transfection. The levels, brightness, and contrast of the panels were adjusted by means of Adobe Photoshop as follows: brightness +120, input level (black) 30, then again brightness +50, contrast +50. Scale bar = 100 µm.

The above viable recombinants R-593, R-613, and R-611 carried the appropriate scFvs in gD, in place of aa 6–38 [27]. As mentioned, the deletion of aa 6–38 ablates targeting and binding to the natural gD receptors HVEM and nectin1 [27]. R-LM249 carries the deletion of amino acids 61–218 in gD, and its replacement with the scFv to HER2 [28]. Here, we engineered the scFv to PSMA, EGFRvIII, or EGFR in place of aa 61–218, thus generating BAC-591, BAC-623, and BAC-621, respectively (Figure 5A,B). When transfected into target cell lines expressing the cognate receptor, the recombinant BAC genomes did not give rise to viable viruses (Figure 5C, panels a, c, e), but only to single EGFP-positive cells. To check whether the recombinant BAC genomes carried defects in genes other than gD, they were transfected in R6 cells, which express wt gD under the UL26.5 promoter [43]. The wt-gD transgenically expressed from these cells provides wt-gD *in trans*, and can pseudotype virions. The results in Figure 5C (panels b, d, f) show that BAC-591, BAC-623, and BAC-621 formed plaques in R6 cells, indicating that there was no defect in their genomes, except the engineered modifications in the gD locus. The pseudotyped viruses were then employed to infect the receptor-positive cognate cells. The recombinants failed to replicate and were lost after the first passage in the respective receptor-positive tumor cells. We interpret the inability of the recombinants generated on the backbone of Δ61–218 gD to grow in the respective target cells as possibly due to substantial alterations in the recombinant gD, leading to loss of function. A non-mutually exclusive possibility is that the constraints exerted by the gD structure may modify the scFv structure, and thus prevent its ability to interact with the cognate receptor. These three recombinants could not be investigated further.

### 3.6. R-291 Carries Mutations in gB Which Increase the Spread in Murine Cells

**Figure 6:**
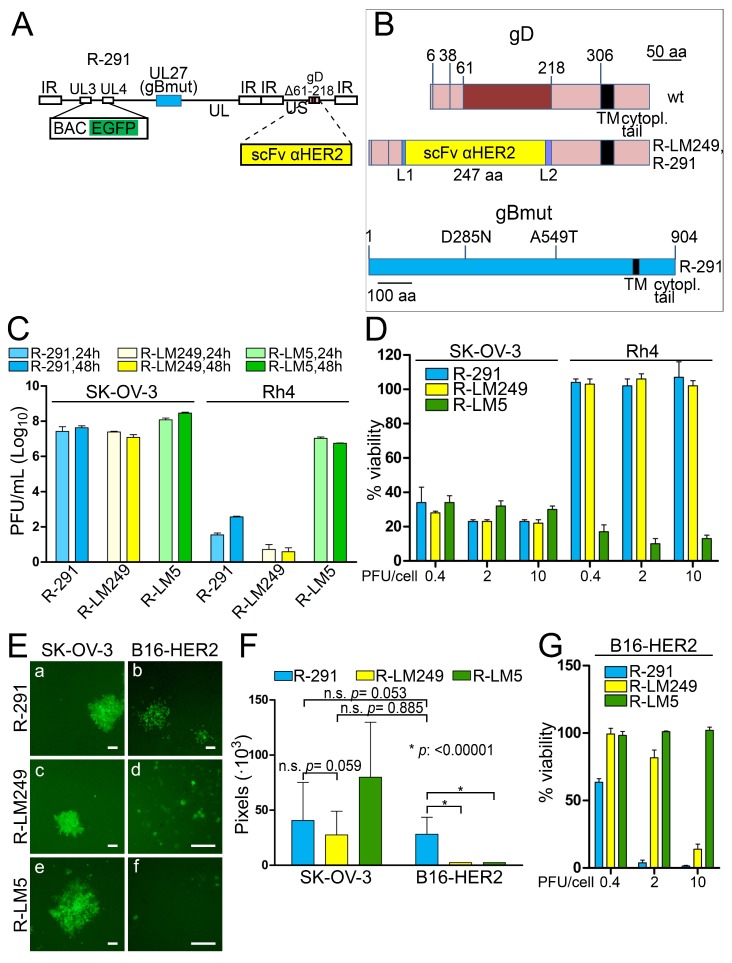
Effect of mutations in gB on virus replication and spread in human and murine cells. (**A**) Schematic drawing of the R-291 genome. The scFv to HER2 is inserted in place of aa 61–218 of gD. pBeloBAC11 and EGFP (BAC EGFP) are inserted between UL3 and UL4 [50]. UL27, encoding gB, is shown as a light blue box. gBmut carries the mutations D285N and A549T, as detailed in panel B. (**B**) Schematic diagram of wt gD (upper line), of R-LM249 and R-291 gD (middle line), and of R-291 gB (bottom line) carrying the mutations D285N and A549T (gBmut). (**C**) Replication of R-291, R-LM249, and R-LM5 recombinants in human HER2-positive (SK-OV-3) or HER2-negative (Rh4) tumor cell lines, infected at 1 PFU/cell, and harvested at 24 and 48 h after infection. The plotted values represent the average of two independent experiments ± SD. (**D**) Effect of virus infection on cell viability. The human HER2-positive (SK-OV-3) or HER2-negative (Rh4) tumor cell lines were infected at the indicated MOI and cell viability was assessed at day 4. The bars represent the averages of at least three replicate samples ± SD. (**E**) Examples of typical plaques by R-291, R-LM249, and R-LM5 in the B16 murine cell line expressing HER2 (B16-HER2), and in SK-OV-3 cells. The levels, brightness, and contrast of the panels were adjusted by means of Adobe Photoshop as follows: (a): input level (black) 30, brightness +50, contrast +50; (b): brightness +50, contrast +80, input level (black) 50; (c) input level (black) 30, brightness +50, contrast +50; (d): input level (black) 30, brightness +50, contrast +50; (e) input level (black) 30, brightness +50, contrast +50; (f) input level (black) 30, brightness +100, contrast +50. Scale bar = 100 µm. (**F**) Average sizes of plaques made by the indicated viruses in B16-HER2, and in the human SK-OV-3 cells, for comparison. The bars represent the average pixel counts for 35 plaques ± SD, in pictures taken with a 4× objective. *: statistically significant; n.s.: not significant. (**G**) Effect of virus infection on B16-HER2 viability. Cells were infected at the indicated MOI and cell viability was assessed at day 4. The bars represent the averages of four replicate samples ± SD.

R-LM249 exhibited a strong in vivo efficacy in immunodeficient mice xenografted with human tumor cells expressing HER2 [28,34]. However, R-LM249 spread poorly in murine cells, thus hampering efforts to evaluate its efficacy in immunocompetent murine models. The D285N and A549T substitutions in HSV gB were described as “hyperactive mutations” capable of increasing HSV-1 cell-to-cell spread and rate-of-entry [59]. A boosted cell-to-cell spread in murine cells would translate into an enhanced efficiency of the oncolytic treatment in an immunocompetent mouse model, where only syngeneic murine cancer cells can be implanted. In addition, mutations which affect the viral rate of entry could increase infectivity and replication in human cells. In order to obtain an HER2-retargeted recombinant more efficient in terms of the infection of murine cancer cells, and, possibly, of human cells, the two gB mutations were inserted in R-LM249 (Figure 6A), thus generating R-291.The linear maps of R-291 gD and gB are shown in Figure 6B. The replication of R-291 in human cancer cell lines, positive or negative for HER2 expression, was compared to that of its parental R-LM249. R-LM5 (wt gD) was included for comparison. Figure 6C shows that R-291 did not exhibit an increase in replication in the HER2-positive SK-OV-3 cells, as compared to R-LM249. R-291 maintained the specific tropism for HER2-positive cells and failed to substantially replicate in the HER2-negative Rh4 cells, similarly to its parent R-LM249. Accordingly, the cytotoxic activities exerted by R-291 and its parent virus R-LM249 for the human SK-OV-3 and Rh4 cells were undistinguishable (Figure 6D). The results on Rh4 cells rule out that the mutations in gB conferred an off-target tropism.

To verify whether the gB mutations conferred an enhanced infection in murine cancer cells, we quantified the average plaque size in a murine cancer cell line stably expressing human HER2 (B16-HER2). Figure 6E, panel b, shows that R-291 formed plaques in B16-HER2 cells, whereas the parent R-LM249 (panel d) and the wt R-LM5 (panel f) only induced singly infected cells. Quantification of the plaque size is reported in Figure 6F. It can be seen that the average plaque size by R-291 in B16-HER2 cells was similar to that reached by R-LM249 in SK-OV-3 cells. Furthermore, the R-291 plaques were similar in size in SK-OV-3 and in B16-HER2 cells. The cell viability assay confirmed that B16-HER2 cells were targeted by R-291, and to a lesser extent by R-LM249 (due to the lack of efficient cell to cell spread), but not by R-LM5. Altogether, the gB mutations improved cell to cell spread among murine cancer cells, but not among the human SK-OV-3 cancer cells. This property may facilitate the analysis of in vivo antitumor efficacy in immunocompetent mice implanted with syngeneic murine cancer cells.

### 3.7. A Novel Insertion Site for Expression of the Heterologous mIL12 Gene

The next aim was to identify an additional insertion site in the HSV genome suitable for the expression of a heterologous gene.

**Figure 7:**
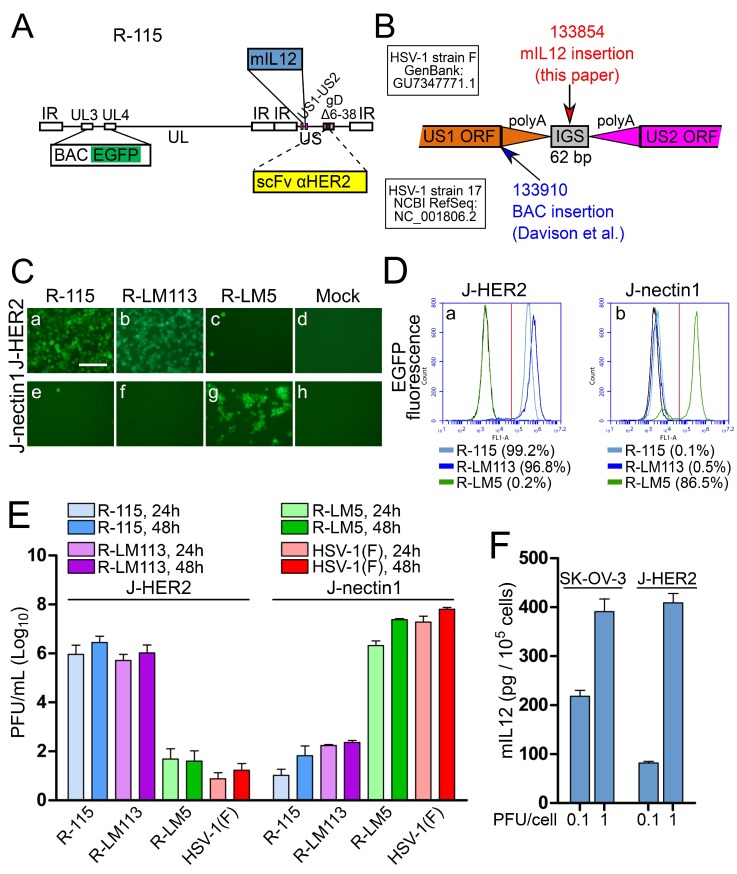
Properties of the HER2-retargeted R-115 expressing mIL12. (**A**) Genome organization or R-115. The scFv to HER2 is in gD, in place of aa 6–38; pBeloBAC11 and EGFP (BAC EGFP) are inserted between UL3 and UL4 [50]; the mIL12 cassette was cloned between US1 and US2. (**B**) Details of the US1-US2 intergenic region. PolyA addition sites are depicted as triangles. The coordinate for HSV-1(F) (GenBank: GU734771.1) and HSV-1 strain 17 (NCBI RefSeq: NC_001806.2) are above and below the diagram of the genes, respectively. The coordinates in red and blue indicate the site of insertion used in this study and by Davison et al. (GenBank: FJ593289.1), respectively. (**C**) J-HER2 or J-nectin1 cells were infected with the indicated viruses at 10 PFU/cell. Pictures were taken at 24 h p.i. The pictures relative to the controls (R-LM5 and Mock in J-nectin1) are the same as shown in Figure 2C and 4C, since the experiments were run in parallel. The levels, brightness, and contrast of the panels were adjusted by means of Adobe Photoshop as follows: input level (black) 30, brightness +60, contrast +60. Scale bar = 100 µm. (**D**) J-HER2 or J-nectin1 cells infected as in panel C and analyzed by flow cytometry for EGFP detection. The controls R-LM5 and Mock (black line) in J-nectin1 were presented in Figure 2D and 4D, as the experiments were run in parallel. The figures next to each virus name indicate the percentage of infected cells. Vertical red line: threshold discriminating the negative and positive populations, as determined by comparison with the negative control. (**E**) R-115 replication in J-HER2 and J-nectin1 cells, infected at 0.1 PFU/cell and harvested at 24 and 48 h after infection. Histograms represent the average of three independent experiments ± SD. (**F**) Quantification of mIL12 secreted in the media of R-115-infected SK-OV-3 or J-HER2 cells, 24 h after infection at 0.1 and 1 PFU/cell. The bars represent the averages of triplicates ± SD.

The known sites in the HSV genome suitable for the insertion of functional transgenes and capable of giving rise to viable recombinants are limited in number. Here, we engineered a cassette for the expression of murine interleukin 12 (mIL12) under the control of pCMV in the US1-US2 intergenic locus of the HER2-retargeted R-LM113, thus generating R-115 (schematic drawing of R-115 genotype in Figure 7A). IL12 is a potent immunostimulatory cytokine, capable of helping to break the tolerance to tumors and induce an immunotherapeutic response [72]. Its systemic administration is marred by toxicity, which can be overcome by loco-regional administration of the cytokine, e.g., by local administration via a viral vector encoding the IL12 gene [73,74,75,76,77,78,79,80]. The rationale for choosing the US1-US2 intergenic locus as the insertion site was as follows. US1-US2 genes are located on opposite strands, but do not overlap, and are separated by an intergenic non coding region containing the two convergent polyA signals and a 62-bp intergenic region (Figure 7B). Previously, this site was described by Cunningham and Davison (GenBank: FJ593289.1) as a site where self-excising BAC sequences were successfully inserted in the DNA of HSV-1 (strain 17). In that example, following the reconstitution of the virus in cell culture, the heterologous sequences were removed. Hence, so far, it was unknown whether this insertion site is suitable for the generation of viable recombinants, for the expression of the inserted gene, and for stable expression. The insertion by Cunningham and Davison was placed immediately downstream of the stop codon of the US1 open reading frame (coordinate 133910 in HSV-1 strain 17, GenBank # NC_001806.2). The insertion separated US1 ORF from its polyA signal sequences (Figure 7B). Different from that construct, in our construct, the mIL12 cassette was engineered at coordinate 133854 (coordinate of HSV-1(F) in GenBank # GU734771.1), i.e., in the central part of the 62-bp intergenic region (133823–133884) (see, schematic drawing in Figure 7B). This insertion preserved the polyA signals for both US1 and US2. Figure 7C–E show that R-115 maintained the same tropism exhibited by its parent R-LM113. As expected, R-115 did not substantially replicate in J-nectin1 cells and replicated to similar titers as R-LM113 in J-HER2 cells (Figure 7E).

To ascertain whether mIL12 was indeed secreted by the infected cells, the medium of SK-OV-3 and J-HER2 cells was harvested 24 h after infection with R-115 (0.1 and 1 PFU/cell). mIL12 was quantified by ELISA, using recombinant mIL12 as standard. The monoclonal employed for mIL12 determination reacted to the C-terminal p35 subunit, and thus ensured recognition of the mIL12 dimer. Figure 7F shows that mIL12 was secreted in the medium of R-115-infected SK-OV-3 and J-HER2 cells, and reached concentrations ranging between 100 and 400 pg/10^5^ cells in the two cell lines at 24 h after infection.

Altogether, the US1-US2 insertion site led to a recombinant capable of expressing the mIL12 transgene.

### 3.8. Detection of Viral Replication through Expression of the Gaussia Luciferase (GLuc) Reporter

**Figure 8:**
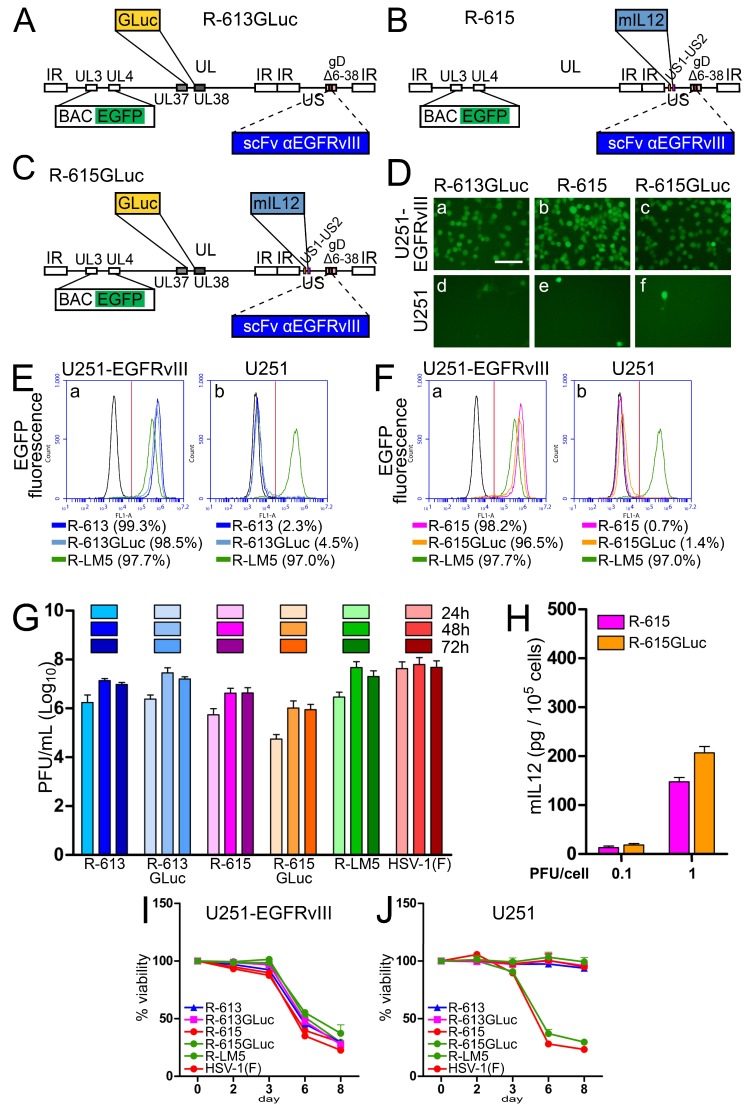
Properties of the EGFRvIII-retargeted recombinants expressing mIL12 and/or GLuc. (**A**–**C**) Schematic drawings of the genomes of recombinants carrying expression cassettes. pBeloBAC11 and EGFP (BAC EGFP) are inserted between UL3 and UL4 [50]. (**A**) R-613GLuc has the same backbone as R-613, i.e., an scFv to EGFRvIII is inserted in place of aa 6–38 of gD. In addition, R-613GLuc carries a cassette for Gaussia Luciferase expression between UL37 and UL38. (**B**) In R-615, the R-613 backbone was engineered with a cassette for the expression of mIL12 between US1 and US2. (**C**) R-615GLuc is R-615 engineered with the cassette for Gaussia Luciferase expression between UL37 and UL38, as in (A). (**D**) U251-EGFRvIII or U251 cells were infected with the indicated viruses at 10 PFU/cell. Pictures were taken at 24 h p.i. The controls (R-LM5 and Mock) and the reference parental virus R-613 are shown in Figure 3C, as the experiments were run in parallel. The levels, brightness, and contrast of the panels were adjusted by means of Adobe Photoshop as follows: input level (black) 30, brightness +60, contrast +60. Scale bar = 100 µm. (**E**,**F**) U251-EGFRvIII or U251 cells mock infected or exposed to 10 PFU/cell of the indicated viruses were analyzed by flow cytometry for EGFP detection at 24 after infection. The samples R-613, R-LM5, and Mock (black line) are the same as shown in Figure 3D, since all the measurements were run in parallel; they are shown here for easiness of comparison. The figures next to each virus name indicate the percentage of infection. Vertical red line: threshold discriminating the negative and positive populations, as determined by comparison with the negative control. (**G**) Replication of R-613GLuc, R-615, and R-615GLuc in U251-EGFRvIII cells, infected at 0.1 PFU/cell, and harvested 24, 48, and 72 h after infection. The virus yield experiment and the titration were carried out simultaneously with those shown in Figure 3E, for easiness of comparison, and therefore the samples relative to R-613, R-LM5, and HSV-1(F) are shared between the experiments. The histogram columns represent the average of three independent experiments ± SD. (**H**) Quantification of mIL12 secreted in the medium of U251-EGFRvIII cells, 24 h after infection with R-615 and R-615GLuc, at 0.1 and 1 PFU/cell. The histogram bars represent the average of triplicates ± SD. (**I**,**J**) Effect of virus infection on cell viability. U251-EGFRvIII (I) or U251 (J) cells were infected with the indicated viruses at 2.5 PFU/cell. The data represent the average of four replicate samples ± SD. The plots of the reference control and parental viruses (R-613, R-LM5 and HSV-1(F)) are the same as shown in Figure 3F,G.

The aim of the next series of experiments was to evaluate whether HSV replication can be monitored through the expression of a recently described reporter, Gaussia Luciferase (GLuc), which can be detected both in culture and in vivo. We engineered two recombinants. The first was R-613GLuc, a derivative of R-613, which carries the GLuc gene in the UL37-UL38 intergenic region (see Figure 8A for genomic organization). The second was R-615GLuc, which simultaneously expresses two transgenes. Preliminarily, we generated R-615, which carries the mIL12 gene in the US1-US2 intergenic region (Figure 8B for genomic organization), and under this respect is therefore similar to R-115. Subsequently, we inserted the GLuc gene in the UL37-UL38 region of R-615, thus generating R-615GLuc (Figure 8C). See Table 1 for summary properties of the recombinants. Figure 8D,F show that the tropism of R-613GLuc, R-615, and R-615GLuc was undistinguishable from that of the parental R-613 (Figure 3C,D). The replication of R-615 and R-615GLuc was somewhat lower than that of R-613 and R-613GLuc (Figure 7G). mIL12 was secreted in the medium of U251-EGFRvIII cells infected with R-615 and R-615GLuc, and at 1 PFU/cell reached overall similar concentrations to those achieved by R-115 in J-HER2 and SK-OV-3 cells (compare Figure 8H with Figure 7F). The recombinants from this family could not be differentiated from each other with respect to the ability to cause cytotoxicity in U251-EGFRvIII cells (Figure 8I).

**Figure 9:**
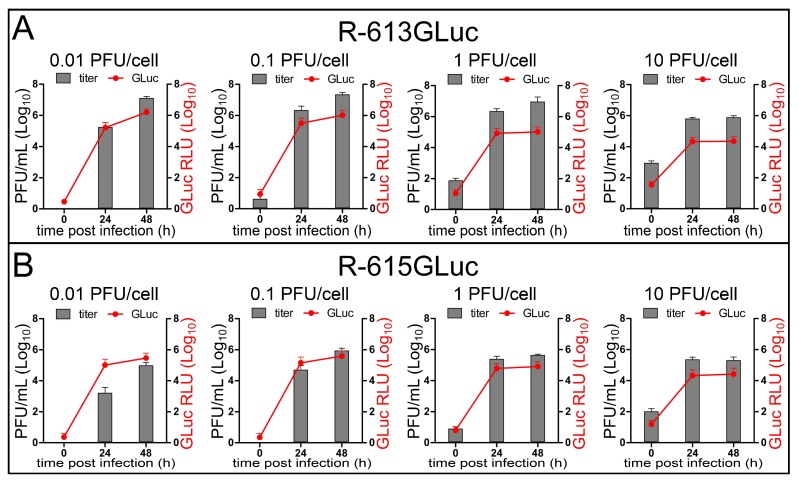
Secretion of GLuc parallels virus replication. Expression of Gaussia Luciferase in U251-EGFRvIII cells infected with R-613GLuc (**A**) or R-615GLuc (**B**) at MOI ranging from 0.01 to 10 PFU/cell. Gaussia Luciferase activity was read from an aliquot of supernatant medium taken from infected cell cultures at the indicated time points, before freezing the samples for total (extra + intra-cellular) virus titer determination. Gaussia Luciferase activity is plotted after normalization to the background of the viral inoculum. Points and bars represent the average of three independent experiments ± SD.

Importantly, Figure 9A,B show that GLuc was secreted from cells infected with R-613GLuc and R-615GLuc. The amount of secreted GLuc paralleled the increase in viral replication.

## 4. Discussion

The next generation oncolytic viruses will likely combine multiple genetic modifications to make the viruses more potent and more cancer-specific, and to achieve the expression of transgenes. In particular, genetic alterations are being designed that act synergistically to target tumors through multiple mechanisms. A strategy towards more potent and more cancer-specific oncolytic viruses is the retargeting of the viral tropism to cancer specific receptors.

In earlier work, we generated two HSV recombinants retargeted to the HER2 tumor cell specific receptor, named R-LM113 and R-LM249. Both are non-attenuated, retargeted to HER2, and detargeted from the natural HSV receptors HVEM and nectin1 [27,28]. Molecularly, the two viruses differ in the gD sequences which were deleted for detargeting purposes, aa 6–38 or aa 61–218 in R-LM113 or R-LM249, respectively. In both viruses, the deleted sequences were replaced with the scFv to HER2 for retargeting purposes. No other genetic modification, including attenuation, was introduced. Hence, they are fully virulent in their target cells. Here, we report that the Δ6–38 deletion in gD (R-LM113 backbone), but not the Δ61–218 deletion in gD (R-LM249 backbone), serves as a platform to engineer HSVs retargeted to the PSMA, EGFRvIII, or EGFR tumor cell specific receptors. We identified a site for transgene insertion which was not verified before for ability to drive the expression of the mIL12 transgene. This transgene was simultaneously expressed with the Gaussia Luciferase reporter. We also found that mutations in gB, known to affect the HSV rate of entry, enhance the HER2-retargeted o-HSV infection and cell to cell spread in murine cells.

### 4.1. Platform to Engineer Novel Retargeted HSVs

The insertion of the specific scFvs in Δ6–38 gD (R-LM113 backbone) resulted in HSV recombinants retargeted to PSMA, EGFRvIII, and EGFR, and detargeted from the natural gD receptors. The recombinants efficiently spread from cell to cell, grew to high titers, and were cytotoxic to cells. Of note was the EGFR-retargeted R-611, which additionally replicated, albeit to a lower extent, in the receptor-negative J cells, as well as in J-nectin1 cells. The latter two infections were inhibited by the anti-EGFR cetuximab, hinting that they occurred through a hamster EGFR ortholog present in J cells. This interpretation is consistent with the finding that R-611 can be grown in RS cells as well. Cumulatively, the current results on R-611 are not in contrast with the conclusions that R-611 is retargeted to EGFR and detargeted from nectin1/HVEM. Rather, they call for a careful evaluation when considering EGFR as a specific objective for targeted therapy. The results with the scFv insertion in the Δ6–38 gD contrast with those obtained with insertion in the Δ61–218 gD (R-LM249 backbone). In the latter case, no viable recombinant could be generated, unless they were pseudotyped with wt gD. Thus, the failure to form infectious virions in the R-LM249 backbone mapped to gD. In conclusion, of four scFvs tested, only the one to HER2 was capable of generating viable recombinants when inserted in the Δ61–218 gD. We note that the scFv to HER2 has the composition V_L_-linker-V_H_, whereas the scFvs to PSMA, EGFRvIII, and EGFR employed in this study had the composition V_H_-linker-V_L_. We also note that when the scFvs were inserted in the Δ6–38 gD (R-LM113 backbone), they were located at the very N-terminus of the chimeric molecule, and most likely were free to adopt their conformation, to position themselves relative to the rest of gD, and let gD do its gymnastics. In contrast, when the scFvs were inserted in the Δ61–218 gD, they replaced the core of the molecule. It is possible that their own conformation was constrained, and/or that the remaining portions of gD, including the profusion domain, were distorted, or were not able to adopt the proper conformation and to carry out the gymnastics required of gD to activate gH and gB.

Importantly, the three recombinants generated in this study were all able to infect human cancer cells expressing the targeted receptors, i.e., PSMA, EGFRvIII, or EGFR. Thus, the level of expression of these receptors in *bona fide* human cancer cells is suitable to enable infection with the retargeted o-HSVs.

### 4.2. Effects of Mutations in gB on Cell-to Cell Spread of Retargeted o-HSVs

The D285N and A549T substitutions in HSV gB described earlier as “hyperactive mutations” [59] conferred to an HER2-retargeted o-HSV an enhanced cell-to-cell spread in B16-HER2 murine cancer cells. Of note, B16 cells are scarcely susceptible to HSV carrying wt gD [63,81]. The boosted cell-to-cell spread in murine cell lines may allow and facilitate the analysis of in vivo antitumor efficacy in immunocompetent mice, which can only accept syngeneic cancer cells. Given that the R-291 tropism to the natural HSV receptors was ablated and the mutations in gB did not enhance the ability of R-291 to spread among HER-2 positive cells, we consider it unlikely that in humans, the gB mutations would expand infection to non-tumor cells.

### 4.3. Functional Insertion of Transgenes in HSV Genome

O-HSVs induce anti-tumor immunity and can be armed with therapeutic transgenes. Indeed, one of the keys to success for the oncolytic HSV Oncovex^GM-CSF^ (T-VEC) was most likely the expression of the GM-CSF transgene. In addition to the insertion of IL12 or GM-CSF, additional cytokines, e.g., IL15; chemokines, e.g., CXCL10; or positive regulators of the immune response, e.g., ligands of co-stimulatory receptors, are being actively investigated [76,82,83,84,85,86]. Expressing them from the viral genome might favor high intratumoral concentrations of the transgenic molecules, and avoid toxicities consequent to systemic delivery. It has thus become crucial to identify additional sites of insertion in the HSV genomes. To our knowledge, sites of insertion which lead to functional transgenic molecules and, at the same time, to viable HSVs capable of strong replication are the intergenic regions between UL3 and UL4 [50], between UL26 and UL27 [87], and between UL37 and UL38 [88]. The intergenic region between US1 and US2 (two non-essential genes in cell culture) was first described in GenBank entry FJ593289.1 (Cunningham and Davison) as a site where self-excising BAC sequences were successfully inserted. In that example, following the reconstitution of the virus in cell culture, the heterologous sequences were removed. Therefore, the effect of insertion at this site on viral replication was not known. At this locus, we inserted mIL12. The resulting recombinants R-115 and R-615 were viable, replicated to high titers, and, to our knowledge, were genetically stable.

The second transgene was the Gaussia Luciferase (GLuc). This reporter was of interest because it is secreted from the cells and its luminescence activity can be measured in extracellular fluids, cell culture medium, or blood, by directly supplying the substrate, without any purification. Quantification of GLuc activity in the blood makes it possible to evaluate virus replication (or alternatively tumor growth) in whole animals by a non-invasive assay [89,90]. In cultured cells infected with two GLuc-expressing recombinants, R-613GLuc and R-615GLuc, the amount of secreted GLuc paralleled the increase in viral replication. It was not possible to unequivocally associate the GLuc level with the viral titer; however, a time-course measurement of GLuc holds promise to be a reliable tool for monitoring viral replication in in vivo experimental settings. The extent of G-Luc expression achieved with R-613GLuc and R-615Gluc (10^8^ relative luciferase units) is much higher than that reported for murine cytomegalovirus (10^4^ relative luciferase units) at an equivalent MOI [90]; the latter virus enabled the evaluation of virus replication in mice in situ and in the blood. Thus, the extent of GLuc expression achieved with the retargeted o-HSVs described here will likely enable the evaluation of virus growth in mice.

## Figures and Tables

**Table 1:** Key features of wt and recombinant (R) viruses, of recombinant viral genomes (BAC), and of the parental viruses employed in this paper.

Recombinant Virus *	Derived From	RETARGETING		ADDITIONAL MODIFICATIONS			Ref.
		Insertion of scFv to **	Insertion in	Genetic Modification ***	Site of Insertion	Purpose	
R-593		PSMA	gD Δ6–38				This paper
R-611		EGFR	gD Δ6–38				This paper
R-613		EGFRvIII	gD Δ6–38				This paper
R-615	R-613	EGFRvIII	gD Δ6–38	mIL12 ****	US1-US2	immunotherapy	This paper
R-613GLuc	R-613	EGFRvIII	gD Δ6–38	GLuc ****	UL37-UL38	luciferase secretion	This paper
R-615GLuc	R-615	EGFRvIII	gD Δ6–38	mIL12 and GLuc	US1-US2 and UL37-UL38	immunotherapy and luciferase secretion	This paper
R-LM113		HER2	gD Δ6–38				[27]
R-115	R-LM113	HER2	gD Δ6–38	mIL12	US1-US2	immunotherapy	This paper
R-LM249		HER2	gD Δ61–218				[28]
R-291	R-LM249	HER2	gD Δ61–218	D285N + A549T in gB	UL27 (gB)	enhanced infection or spread	This paper
Recombinant viral genome *							
BAC-591		PSMA	gD Δ61–218				This paper
BAC-621		EGFR	gD Δ61–218				This paper
BAC-623		EGFRvIII	gD Δ61–218				This paper
		**NO RETARGETING**					
**Wt-virus and recombinant**		**Natural receptors**					
HSV-1(F)		Nectin1, HVEM					[49]
R-LM5	HSV-1(F)	Nectin1, HVEM		BAC sequences	UL3-UL4	Genetic engineering in bacteria	[27]

All the recombinants derive from a backbone carrying pBeloBAC11 and EGFP inserted between UL3 and UL4 [27,50]. * Prefix R- denotes recombinant virus; prefix BAC denotes recombinant viral genome, as BAC-DNA. ** Size of inserts ranges between 747 and 801 bp, including flanking serine-glycine linkers upstream and/or downstream the scFv. *** Size of inserts: 1326 bp (GLuc cassette), 3200 bp (mIL12 cassette). **** mIL12 (murine interleukin 12); GLuc (Gaussia Luciferase).

**Table 2:** Cell lines employed for the characterization of the recombinant viruses and BACs.

Cell Line	Infecting Recombinant Virus	Displayed Receptor	Genetic Modification	Tissue/Cell Type	Ref.
**Human cancer**					
LNCaP	R-593, R-LM5	Natural HSV receptors *, PSMA	none	hu prostate adenocarcinoma	[51]
MDA-MB-231	R-LM5, R-611	Natural HSV receptors *, EGFR	none	hu mammary adenocarcinoma	[52]
hGic-G7	R-LM5	Natural HSV receptors *	none	hu glioblastoma	[45]
hGic-G15	R-613, R-LM5	Natural HSV receptors *, EGFRvIII	none	hu glioblastoma	[45]
PC3-PIP-PSMA	R-593	Natural HSV receptors *, PSMA	express hu PSMA	hu prostate adenocarcinoma	[53]
Rh4	R-LM5	Natural HSV receptors *	none	hu muscle rhabdomyosarcoma	[44]
SK-OV-3	R-LM5, R-LM113, R-115, R-LM249, R-291, R-611	Natural HSV receptors *, HER2, EGFR	none	hu ovary adenocarcinoma	[54]
U251**	R-LM5	Natural HSV receptors *, EGFR	none	hu glioma	[55]
U251-EGFRvIII	R-613, R-615, R-613GLuc, R-615GLuc, R-LM5	Natural HSV receptors *, EGFR, EGFRvIII	express hu EGFRvIII	hu glioma	This paper
**Animal cell lines**					
B16-HER2	R-LM249, R-291	HER2	express hu HER2	mu melanoma	[48]
CHO-EGFR	R-611	EGFR	express hu EGFR	hamster ovary	[46]
J	none	none	wt HSV receptor negative	hamster kidney	[47]
J-EGFR	R-611	EGFR	express hu EGFR	hamster kidney	This paper
J-EGFRvIII	R-613, R-615, R-613GLuc, R-615GLuc	EGFRvIII	express hu EGFRvIII	hamster kidney	This paper
J-HER2	R-LM113, R-115, R-LM249, R-291	HER2	express hu HER2	hamster kidney	[27]
J-nectin1	R-LM5	Nectin1	express hu nectin1	hamster kidney	[47]
J-PSMA	R-593	PSMA	express hu PSMA	hamster kidney	This paper
RS	R-611, R-LM5	not characterized***	none	rabbit skin	
R6	BAC-591, BAC-621, BAC-623, R-593, R-611, R-613	not characterized***	inducible HSV gD	rabbit skin	[43]
TRAMP-PSMA	R-593	PSMA	express hu PSMA	mu prostate epithelium	[56]

* Nectin1, HVEM/HveA; ** U251MG: for simplicity “U251” throughout the paper; *** can be inferred from range of infection.

**Table 3:** Cell lines employed for titration of the viruses used in this paper.

Cell line Employed for Titration	Virus
SK-OV-3	HSV-1(F), R-LM5, R-LM113, R-115, R-LM249, R-291, R-611
J-PSMA	R-593
U251-EGFRvIII	R-613, R-615, R-613GLuc, R-615GLuc

**Table 4:** Templates and primers for the second step of galK recombineering.

Recombinant	Template	Forward Primer	Reverse Primer
BAC-291	gB_D285N_A549T cl 2	gBint4: GGGCATCGCGGTGGTCTTCAAGGAGAACA	gB_2046_r: GCGGGTGTACACCTCCAGGGGGACAAAC
BAC-591	pSL1180-scFv-PSMA	gD60 + 8SG_J591_f: GCCTCCCGATCACGGTTTACTACGCCCATAGTAGTGGCGGTGGCTCTGGAGAGGTGCAGCTGCAGCAGTCAGGACC	12SG + gD219_J591_r: AAGCGGGGCAGCATACCGGAtCcACCGGAACCAGAGCCACCGCCACTCGACCGTTTCAGGTCCAGCATGGTCCCAG
BAC-593	BAC-591	gD5_J591_f: TTGTCGTCATAGTGGGCCTCCATGGGGTCCGCGGCAAATATGCCTTGGCGGAGGTGCAGCTGCAGCAGTCAGGACC	gD39_SG11_r: ATCGGGAGGCTGGGGGGCTGGAACGGGTCCGGTAGGCCCGCCTGGATGTGGGATCCACCGGAACCAGAGCCACCGC
BAC-611	pTNHaa-EGFR	BAC_LM611_f: TTGTCGTCATAGTGGGCCTCCATGGGGTCCGCGGCAAATATGCCTTGGCGGCCGAGGTGCAACTGCAGCAGTC	BAC_LM611_r: AGGCCCGCCTGGATGTGGGATCCACCGGAACCAGAGCCACCGCCACTCGATTTGATCTCGAGTTCTGTCCCCG
BAC-613	pMR1ENV1	BAC_LM613_f: TTGTCGTCATAGTGGGCCTCCATGGGGTCCGCGGCAAATATGCCTTGGCGCAGGTGAAACTGCAGCAGTCTGG	BAC_LM613_r: AGGCCCGCCTGGATGTGGGATCCACCGGAACCAGAGCCACCGCCACTCGATTTGATTTCCAGCTTGGTGCCATCACC
BAC-621	pTNHaa-EGFR	BAC_LM621_f: GCCTCCCGATCACGGTTTACTACGCCCATAGTAGTGGCGGTGGCTCTGGAGCCGAGGTGCAACTGCAGCAGTC	BAC_LM621_r: AAGCGGGGCAGCATACCGGATCCACCGGAACCAGAGCCACCGCCACTCGATTTGATCTCGAGTTCTGTCCCCG
BAC-623	pMR1ENV1	BAC_LM623_f: GCCTCCCGATCACGGTTTACTACGCCCATAGTAGTGGCGGTGGCTCTGGACAGGTGAAACTGCAGCAGTCTGG	BAC_LM623_r: AAGCGGGGCAGCATACCGGATCCACCGGAACCAGAGCCACCGCCACTCGATTTGATTTCCAGCTTGGTGCCATCACC
BAC-115, BAC-615	pLM84	US1/US2_pCMV_f: ATGTCCCCAAATAAAAGACCAAAATCAAAGCGTTTGTCCCAGCGTCTTAATGGCGGGAAGCGTTTTGCGCTGCTTCGCGATGTACGGGC	US1/US2_polyA_r: CCCCGATGTCAATAAACCCCCAAACACCCCCCATGTACGCGTGGTCTGTTTCTCTCCGCCGCCATAGAGCCCACCGCATCCCCAGCATGCCTG
BAC-613GLuc, BAC-615GLuc	pCMV-GLuc2	UL37/38_pcmv(gau)_f: CCGCAGGCGTTGCGAGTACCCCGCGTCTTCGCGGGGTGTTATACGGCCACCGATGTACGGGCCAGATATACGCGTTGAC	UL37/38_polyA(gau)_r: TCCGGACAATCCCCCGGGCCTGGGTCCGCGAACGGGATGCCGGGACTTAACACACAAAAAACCAACACACAGATGTAATG

## Supplementary Materials

The following are available online at http://www.mdpi.com/1999-4915/10/7/352/s1.
