# Supplementary material for: HSV as A Platform for the Generation of Retargeted, Armed, and Reporter-Expressing Oncolytic Viruses
Source: Viruses. 2018 Jun 30;10(7):352. doi: 10.3390/v10070352 (PMC6070899; doi:10.3390/v10070352)
Supplement: Supplementary file 1 [file viruses-10-00352-s001.pdf]

viruses

## 1. Supplementary Material and Methods

### *1.1. Viral tropism*

Cell monolayers were exposed to the recombinant viruses and control wt-gD viruses R-LM5 and HSV-1(F) at a multiplicity of infection of 10 PFU/cell. Flow cytometry (BD Accuri) was used to measure the binding of MAb 52S to gH followed by an anti-mouse allophycocyanin (APC)-conjugated secondary antibody.

### *1.2. Real time RT-PCR quantification of EGFRvIII*

Total RNA was extracted from hGic-G7 and hGic-G15 using the RNAeasy Kit (Qiagen). Retrotranscription of total RNA was carried out with iScript RT (Biorad), and real-time PCR was carried out with iQ™ SYBR® Green Supermix (Biorad) using the following primers: EGFRvIII-forward (GGC TCT GGA GGA AAA GAA AGG TAA T); EGFRvIII-reverse (TCC TCC ATC TCA TAG CTG TCG); hL41- forward (AGT GGA GGA AGA AGC GAA TG); and hL41-reverse (TTT ATG AGC AAG GTG GGT CTC).

## 2. Supplementary Results

### *2.1. Viral tropism*

The tropism of the recombinant viruses generated in this study retargeted to PSMA, EGFRvIII, EGFR, and expressing the mIL12 cytokine and/or GLuc reporter, was monitored by flow cytometry as EGFP fluorescence as stated in the main text, and as gH expression. The Supplementary Table reports the mean fluorescence values of cells exposed to the virus at 10 PFU/cell and stained at 24 h p.i. with a MAb to gH followed by a secondary antibody.

**Supplementary Table.** gH expression (mean fluorescence) in cell lines mock infected or exposed to 10 PFU/cell of wt HSV-1(F) or the recombinant viruses described in this paper.

| Virus | Cell line | | | | | |
|---|---|---|---|---|---|---|
| | **J-Nectin1** | **J-PSMA** | **J-EGFR** | **J-HER2** | **U251** | **U251-EGFRvIII** |
| HSV-1(F) | 7042.20 | 1701.32 | 1565.69 | 1499.49 | 29330.16 | 11135.60 |
| R-LM5 | 6188.10 | 1766.39 | 1763.64 | 1611.70 | 15179.83 | 16535.06 |
| R-593 | 2282.53 | 9139.41 | | | | |
| R-611 | 2014.27 | | 4903.12 | | | |
| R-613 | | | | | 2192.77 | 17747.63 |
| R-613GLuc | | | | | 2181.21 | 21539.39 |
| R-615 | | | | | 1951.97 | 18886.05 |
| R-615GLuc | | | | | 2247.38 | 18322.34 |
| R-LM113 | 3320.75 | | | 9291.38 | | |
| R-115 | 2154.89 | | | 11221.58 | | |
| Mock | 1740.28 | 1766.08 | 1607.03 | 1807.61 | 1633.82 | 1870.98 |

### *2.2. Expression of EGFRvIII in human glioblastoma initiating cells (hGics)*

We quantified the expression of EGFRvIII in human glioblastoma initiating cells hGic-G7 and hGic-G15 by real time RT-PCR. Expression was at least 250 fold higher in hGic-G15 than in hGic-G7, where it was undetectable over 40 PCR cycles; hence hGic-G7 and hGic-G15 were considered to be negative and positive for EGFRvIII, respectively (Figure S1).

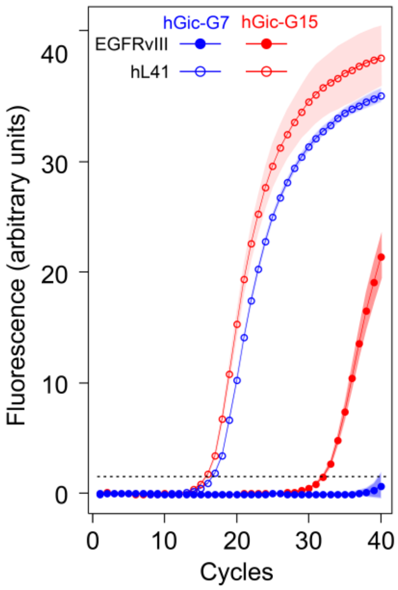


**Figure S1.** Expression of EGFRvIII in human glioblastoma initiating cells. Real-time RT-PCR run on total RNA extracted from hGic-G7 (blue) and hGic-G15 (red) for EGFRvIII expression (full symbols). The hL41 housekeeping gene was included as a positive control (open symbols).
